# Effects of circFOXO3 on the Proliferation and Invasion of Liver Cancer Cells by Regulating PI3K/Akt Pathway

**DOI:** 10.1155/2022/2109908

**Published:** 2022-07-14

**Authors:** Siyu Liu, Minzi Yao, Xiaolong Li, Yumei Liu, Cuiling Xie

**Affiliations:** Department of General Surgery, The First Affiliated Hospital of Shaoyang University, Shaoyang 422000, China

## Abstract

**Objective:**

Hepatocellular carcinoma is a malignant disease occurring in the liver and is one of the main causes of death in cancer patients. Tumor cells are the main components of tumors and have a strong proliferative capacity. They are easily transferred to other parts of the body and can produce harmful substances that destroy the normal organ structure and endanger human life and health. In this study, we investigate the effect of circFOXO3 on the proliferation and invasion of hepatocellular carcinoma cells and its possible mechanism.

**Methods:**

Human hepatocellular carcinoma cells BEL-7404, Hep G2, Hep 3B2.1–7, HuH-7, Li-7, and human normal hepatocytes HHL-5 were selected, and the expression level of circFOXO3 in the cell lines was determined by qRT-PCR. The cell line with low circFOXO3 expression level (HuH-7 cells) was used for follow-up experiments. HuH-7 liver cancer cells were divided into the control group (normal cultured), circFOXO3-NC group (transfected with circFOXO3 negative control), circFOXO3 mimic group (transfected with circFOXO3 mimic), PI3K activator group (20 *μ*mol/L PI3K activator 740Y-P), and circFOXO3 mimic + PI3K activator group (transfected with circFOXO3 mimic + treated with PI3K activator 740Y-P). The qRT-PCR method was used to determine the expression level of circFOXO3 in HuH-7 liver cancer cells in each group, WB was used to detect the expression of apoptosis, invasion, and phosphoinositide 3-kinase/protein kinase B (PI3K/Akt) pathway related proteins in HuH-7 liver cancer cells in each group, the CCK-8 method was used to determine the viability of HuH-7 liver cancer cells in each group, flow cytometry was used to determine the apoptotic ability of HuH-7 liver cancer cells in each group, the transwell chamber experiment was used to determine the invasion ability of HuH-7 liver cancer cells in each group, and the scratch test was used to determine the migration ability of HuH-7 liver cancer cells in each group.

**Results:**

circFOXO3 showed low expression in liver cancer cells; compared with the control group, the circFOXO3 expression and apoptosis rate of HuH-7 liver cancer cells in the circFOXO3 mimic group were significantly increased (*P* < 0.05) and the PI3K/Akt pathway-related protein expression, cell viability, invasion, and migration abilities were significantly reduced (*P* < 0.05); the apoptosis rate of HuH-7 liver cancer cells in the PI3K activator group was significantly reduced (*P* < 0.05) and the PI3K/Akt pathway related protein expression, cell viability, invasion and migration abilities were significantly increased (*P* < 0.05). Compared with the circFOXO3 mimic group, the apoptosis rate of HuH-7 liver cancer cells in the circFOXO3 mimic + PI3K activator group was significantly reduced (*P* < 0.05) and the PI3K/Akt pathway-related protein expression, cell viability, invasion and migration abilities were significantly increased (*P* < 0.05).

**Conclusion:**

Highly expressed circFOXO3 can inhibit the proliferation and invasion of HuH-7 liver cancer cells, which may be achieved by inhibiting the PI3K/Akt pathway.

## 1. Introduction

Liver cancer is a malignant tumor disease occurring in the liver, and it is one of the main causes leading to the death of cancer patients. Virus infection, alcohol, environment, dietary habits, and other factors are all related to the occurrence of liver cancer. Patients with liver cancer often suffer from adverse symptoms such as pain, emaciation, hepatomegaly, and fatigue. Complications such as liver failure, rupture bleeding, and gastrointestinal bleeding will occur in the later stage, which seriously affects the life safety of patients [1, 2]. Tumor cells are the main components of tumors, which have strong proliferation ability. They are easy to transfer to other parts of the body and will produce harmful substances damaging the normal organ structure and endangering human life and health. Therefore, the study of the possible mechanism of tumor cell proliferation and invasion plays an important role in inhibiting the occurrence and development of cancer clinically [3].

Circular RNA (circRNA) is a special kind of noncoding RNA, which is abundant and stable in eukaryotes and participates in many biological activities such as protein synthesis, gene expression, and posttranscriptional modification. circFOXO3 is a member of circRNA family, which is closely related to tumor occurrence, cell survival, and apoptosis [4]. Recent studies have shown that the low expression level of circFOXO3 in breast cancer [5] and esophageal squamous cell carcinoma [6] is closely related to the occurrence and development of tumor diseases. The phosphatidylinositol 3 kinase/protein kinase B (PI3K/Akt) signaling pathway plays an important role in the proliferation and migration of various tumor cells [7]. Some studies have found that downregulating the expression level of PI3K/Akt pathway-related proteins can significantly reduce the proliferation and migration ability of hepatoma cells [8].

Therefore, highly expressed circFOXO3 can inhibit the proliferation and invasion of HuH-7 liver cancer cells, which can be achieved by inhibiting the PI3K/Akt pathway. The Akt pathway is achieved. In this study, by measuring the transfection of circFOXO3 mimicking liver cancer cells, we explored the effect of circFOXO3 on the proliferation and migration of liver cancer cells and its possible mechanism, thus providing new ideas for clinical remission and treatment of liver cancer. Therefore, highly expressed circFOXO3 can inhibit the proliferation and invasion of HuH-7 liver cancer cells, which can be achieved by inhibiting the PI3K/Akt pathway. The Akt pathway is achieved. In this study, by measuring the transfection of circFOXO3 mimicking liver cancer cells, we explored the effect of circFOXO3 on the proliferation and migration of liver cancer cells and its possible mechanism, thus providing new ideas for clinical remission and treatment of liver cancer.

## 2. Materials

### 2.1. Cells

Human hepatoma cells BEL-7404, Hep G2, Hep 3B2.1–7, HuH-7, and Li-7(CL-0032, CL-0103, CL-0102, CL-0120, and CL-0139) were purchased from Wuhan Procell Life Science and Technology Co., Ltd.; human normal hepatocyte HHL-5 (item number: BFN6072012687) was purchased from Shanghai Cell Bank.

### 2.2. Main Reagents and Instruments

740 Y-P (PI3K activator, item number: Hy-P0175) was purchased from MedChemExpress; RNA extraction kit and reverse transcription kit (item number: R1200, T2240) were purchased from Beijing Solarbio Technology Co., Ltd.; protein extraction kit (RIPA lysate) and protein concentration determination kit (item number: P0013B, P0012S) were purchased from Shanghai Beyotime Biotechnology; EntransterTM-R4000RNA transfection reagent (item number: 4000–3) was purchased from Beijing Engreen Biosystem Co., Ltd.; Caspase-3, Bcl-2, Bax, MMP-2, MMP-9, PI3K, p-PI3K, Akt, p-Akt, GAPDH primary antibody, and goat anti-rabbit IgG secondary antibody (item number: ab184787, ab194583, ab32503, ab181286, ab76003, ab32089, ab27854, ab8805, ab38449, ab9485, and ab150077) were purchased from British Abcam.

Cell incubators (model: BC-J80/160S) were purchased from Shanghai Boxun Medical Bioinstrumentation Co., Ltd.; PCR instrument (model: PikoReal) was purchased from Thermo Scientific, USA; cell counters (model: NucleoCounter NC-250 TM) were purchased from ChemoMetec, Denmark; multifunctional microplate readers (model: SpectraMax iD3) were purchased from Molecular Devices; inverted microscopes (model: Ti2 Ts2) were purchased from Nikon, Japan; Gel imagers (model: SmartGel6000) were purchased from Beijing Saizhi Pioneering Technology Co., Ltd.; flow cytometers (model: CytoFLEX) were purchased from Beckman Coulter, Inc., USA.

## 3. Methods

### 3.1. Cell Culture

**Figure 1:**
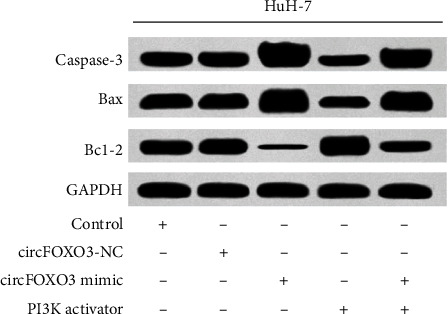
Apoptosis and expression level of apoptosis-related proteins of HuH-7 hepatoma cells in each group.

Human hepatocarcinoma cells BEL-7404, Hep G2, Hep 3B2.1–7, HuH-7, Li-7, and normal human hepatocytes were thawed quickly and cultured in RPMI 1640 medium containing 10% fetal bovine serum and 1% streptomycin at 37°C in a 5% CO_2_ incubator, as shown in Figure 1.

### 3.2. Assay of circFOXO3 mRNA Expression Level in Hepatoma Cells

**Figure 2:**
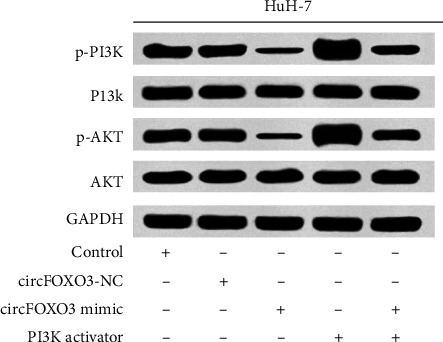
The expression level of PI3K/Akt pathway-related proteins in HuH-7 hepatoma cells in each group.

Human hepatoma cells BEL-7404, Hep G2, Hep 3B2.1–7, HuH-7, and Li-7 and normal human liver cell strains were collected. Total RNA was extracted from the cells by using an RNA extraction kit. cDNA was obtained by using reverse transcription RNA and amplified by PCR. Upstream primer of circFOXO3: 5′-GTGGGGAACTTCACTGGTGCTAAG-3'; downstream primer: 5′-GGGTTGATGATCCACCAAGAGCTCTT-3'; using U6 asinternal reference, upstream primer: 5′-CTCGCTTCGGCAGCACA-3′, downstream primer: 5′-AACGCTTCACGAATTTGCGT-3'. The expression level of circFOXO3 in human hepatoma cells and normal human hepatocytes was calculated by the 2^−∆∆CT^ algorithm, as shown in Figure 2.

### 3.3. Grouping and Transfection of HuH-7 Hepatoma Cells

The HuH-7 hepatoma cells were divided into the control group (normal culture), circFOXO3-NC group (negative control transfected with circFOXO3), circFOXO3 mimetic group (mimetic transfected with circFOXO3), PI3K activator group (20 *µ*mol/L PI3K activator 740Y–P) [9], and circFOXO3 mimic + PI3K activator group (treatment of mimetic transfected with circFOXO3 + PI3K activator 740Y–P). The assay of the expression level of circFOXO3 in HuH-7 hepatocarcinoma cells in each group was the same as that in Section 2.2.

### 3.4. Assay of Vitality and Apoptosis Ability of HuH-7 Hepatoma Cells in Each Group

The transfected HuH-7 hepatoma cells in each group were prepared into cell suspension and then inoculated in 96-well plates (1500 cells/well). The cell viability of HuH-7 hepatoma cells in each group was determined by the CCK-8 kit. For specific operations, refer to the instructions of the CCK-8 kit.

**Figure 3:**
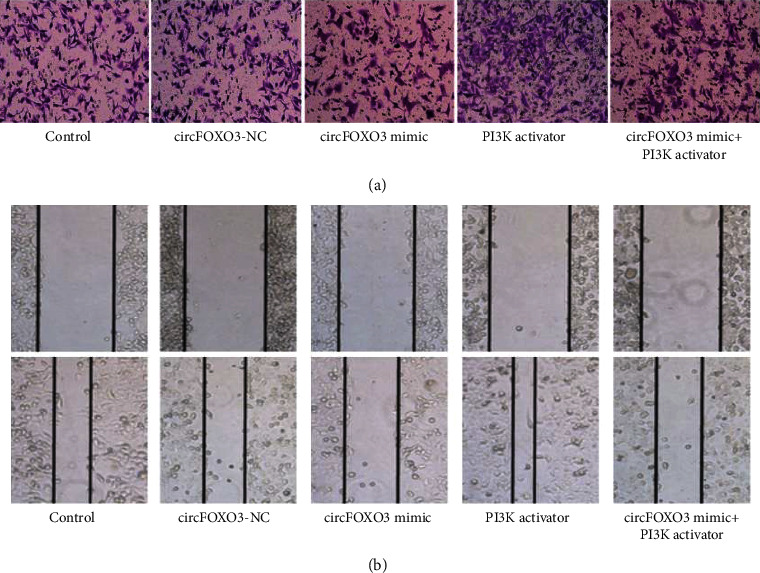
(a) Invasion of HuH-7 hepatoma cells in each group (×200). (b) Migration of HuH-7 hepatoma cells in each group.

The transfected HuH-7 hepatoma cells of each group were collected. 100 *μ*L of cell suspension with the concentration of 1 × 10^6^ cells/mL was added into the flow tubes, and the apoptosis rate of HuH-7 hepatoma cells of each group was measured by using the Annexin V-Alexa Fluor 647/PI apoptosis kit. For specific operations, refer to the instructions of the apoptosis kit (Figure 3).

### 3.5. Assay of Invasion and Migration Abilities of HuH-7 Hepatoma Cells in Each Group

Transfected HuH-7 hepatoma cells of each group were taken, and the invasion ability of cells was determined by the transwell chamber experiment. Matrigel glue was laid in the transwell chamber. The culture medium containing fetal bovine serum was added in the lower chamber, and the HuH-7 hepatoma cell suspension of each group was added in the upper chamber with a concentration of 1 × 10^5^ cells/mL. After culturing in an incubator for 24 h, the residual HuH-7 hepatoma cells in the upper chamber were wiped off. After that, the chamber was fixed with paraformaldehyde and stained with crystal violet. After drying, the cells were observed under a microscope and the number of invasive cells was calculated.

A scratch test was adopted to measure the cell migration ability. The transfected HuH-7 hepatoma cells were inoculated in 6-well plates and cultured in the incubator for 24 h. Vertical scratches were made on the bottom of the well plate with a gun head. Then, the 6-well plates were cultured in an incubator for another 24 h. The scratch healing rate was calculated. The higher the scratch healing rate is, the stronger the cell migration ability is. Scratch healing rate (%) =  (0 h scratch width −24 h scratch width)/0 h scratch width × 100%.

### 3.6. Assay of PI3K/Akt Pathway-Related, Apoptosis-Related, and Invasion-Related Proteins in HuH-7 Hepatoma Cells of Each Group

The transfected HuH-7 hepatoma cells of each group were taken. The total protein in the cells to be detected was extracted by the protein extraction kit and the protein content was determined. The protein was separated by SDS polyacrylamide gel electrophoresis. The separated protein was transferred to the PVDF membrane by the wet transfer method and then sealed by adding 5% skimmed milk powder for 1 h. After incubation at low temperature overnight, horseradish peroxide labeled goat anti-rabbit IgG secondary antibody was added for incubation for another 2 h. Color development was carried out with an enhanced chemiluminescence reagent. Relative expression levels of PI3K/Akt pathway-related, apoptosis-related, and invasion-related proteins in HuH-7 hepatoma cells of each group were analyzed by Image J software.

### 3.7. Statistical Analysis

The experimental data were statistically analyzed by SPSS 22.0 software, and the measurement data were expressed by mean ± standard deviation ($\bar{x}\pm s$). One-way ANOVA was used for comparison between the two groups, and the LSD-t test was used for further comparison between the two groups. *P* < 0.05 indicated that the difference was statistically significant.

## 4. Results

### 4.1. Comparison of circFOXO3 mRNA Expression Level in Hepatoma Cells

Compared with human normal hepatocyte HHL-5, the expression level of circFOXO3 mRNA in human hepatoma cells BEL-7404, Hep G2, Hep 3B2.1–7, HuH-7, and Li-7 significantly decreased (*P* < 0.05). Among them, the expression level of circFOXO3 in HuH-7 cells was low, so HuH-7 cells were selected for subsequent experiments. There was no significant difference between the circFOXO3-NC group and the PI3K activator group. HuH-7 liver cancer cell viability and Bcl-2 protein expression level were significantly increased. The expression levels of asparaginase-3 and Bax proteins were significantly decreased. The viability and Bcl-2 protein expression levels of HuH-7 liver cancer cells in the circFOXO3 simulation group were significantly decreased, while the apoptosis rate, Caspase-3, and Bax protein expression were significantly increased (*P* < 0.05), as given in Table 1.

### 4.2. Comparison of circFOXO3 Expression Level in HuH-7 Hepatoma Cells in Each Group

Compared with the control group, there was no significant difference between the circFOXO3-NC group and the PI3K activator group (*P* > 0.05), but the expression level of circFOXO3 in the circFOXO3 mimic group and circFOXO3 mimic + PI3K activator group increased significantly (*P* < 0.05), as given in Table 2.

### 4.3. Comparison of Vitality and Apoptosis Ability of HuH-7 Hepatoma Cells in Each Group

Compared with the control group, the circFOXO3-NC group had no significant difference (*P* > 0.05). In the PI3K activator group, the vitality and Bcl-2 protein expression level of HuH-7 hepatoma cells increased significantly (*P* < 0.05), while the apoptosis rate and Caspase-3 and Bax protein expression levels of hepatoma cells decreased significantly (*P* < 0.05). The vitality and Bcl-2 protein expression level of HuH-7 hepatoma cells decreased significantly (*P* < 0.05), while the apoptosis rate, Caspase-3, and Bax protein expression increased significantly (*P* < 0.05) in the circFOXO3 mimic group. Compared with the circFOXO3 mimic group, the vitality and Bcl-2 protein expression level of HuH-7 hepatoma cells in the circFOXO3 mimic + PI3K activator group significantly increased (*P* < 0.05), while the apoptosis rate, Caspase-3, and Bax protein expression level of hepatoma cells significantly decreased (*P* < 0.05), as given in Table 3.

### 4.4. Comparison of PI3K/Akt Pathway-Related Protein Expression in HuH-7 Hepatoma Cells in Each Group

Compared with the control group, there was no significant difference in the circFOXO3-NC group (*P* > 0.05). The expression levels of p-PI3K and p-Akt protein in HuH-7 hepatoma cells in the PI3K activator group significantly increased (*P* < 0.05), while the expression levels of p-PI3K and p-Akt protein in HuH-7 hepatoma cells in the circFOXO3 mimic group significantly decreased (*P* < 0.05). Compared with the circFOXO3 mimic group, the expression levels of p-PI3K and p-Akt protein in HuH-7 hepatoma cells in the circFOXO3 mimic + PI3K activator group significantly increased (*P* < 0.05), as given in Table 4.

### 4.5. Comparison of Invasion and Migration Ability of HuH-7 Hepatoma Cells in Each Group

Compared with the control group, the circFOXO3-NC group had no significant difference (*P* > 0.05). The invasion and migration ability and MMP-2 and MMP-9 protein expression levels of HuH-7 hepatoma cells in the PI3K activator group significantly increased (*P* < 0.05), while the invasion and migration ability of MMP-2 and MMP-9 protein expression levels of HuH-7 hepatoma cells in the circFOXO3 mimic group significantly increased. Compared with the circFOXO3 mimic group, the invasion and migration ability and MMP-2 and MMP-9 protein expression levels of HuH-7 hepatoma cells in the circFOXO3 mimic + PI3K activator group significantly increased (*P* < 0.05), as given in Table 5.

## 5. Discussion

Patients with liver cancer are often treated by surgical resection, radiotherapy and chemotherapy, chemotherapy, and biotherapy, but the overall mortality remains high [10, 11]. Malignant proliferation, strong invasion, and migration ability of hepatoma cells are the premise and foundation of tumor diffusion and metastasis, as well as one of the main reasons for further development of tumor disease [12]. Therefore, the exploration of the related mechanism of proliferation and invasion of hepatoma cells has a positive effect on clinical inhibition and alleviation of liver cancer.

Widely existing in eukaryotes, circRNA has certain regulatory effects on protein synthesis, gene expression, transcription modification, and other cellular biological functions and plays an important role in the occurrence and development of various tumor diseases. It is one of the latest research hotspots in the field of RNA [13]. circFOXO3, a member of the circRNA family, has low expression in nonsmall cell lung cancer [14] and esophageal squamous cell carcinoma [15], closely related to biological activities such as tumor cell proliferation and migration. Wang [16] and other studies found that the overexpression of circFOXO3 can inhibit the proliferation of esophageal squamous cell carcinoma and promote the apoptosis of esophageal squamous cell carcinoma. The results of this study showed that the expression level of circFOXO3 in hepatoma cells BEL-7404, Hep G2, Hep 3B2.1–7, HuH-7, and Li-7 decreased significantly, which suggested that circFOXO3 was closely related to the occurrence and development of liver cancer. Besides, the expression level of circFOXO3 in HuH-7 hepatoma cells was low. Thus, HuH-7 hepatoma cells were selected in a follow-up study for the transfection experiment. The results showed that the activity, invasion, and migration ability of hepatoma cell HuH-7 decreased significantly and the apoptosis rate increased significantly after increasing the expression level of circFOXO3 in hepatoma cells, which indicated that the highly expressed circFOXO3 could inhibit the proliferation and migration abilities of hepatoma cells and had a certain anticancer effect. The results were consistent with the above research.

The PI3K/Akt signaling pathway is closely related to cell proliferation, apoptosis, metastasis, and other activities. Akt is an important downstream signaling molecule of PI3K, and PI3K mainly participates in the regulation of cell proliferation, apoptosis, and other signal transduction by activating Akt [17]. Recent studies have found that the PI3K/Akt signaling pathway plays an important role in the proliferation and migration of hepatoma cells [18]. Inhibition of the PI3K/Akt signaling pathway and reduction of phosphorylation of PI3K and Akt can inhibit the proliferation and migration of hepatoma cells and promote the apoptosis of hepatoma cells [18–20]. The expression level of PI3K/Akt pathway-related proteins in HCC cells in the circFOXO3 mimic + PI3K activator group was significantly higher than that in the circFOXO3 mimic group, suggesting that the overexpression level of circFOXO3 could inhibit the proliferation and migration of HCC cells by inhibiting the PI3K/Akt pathway.

To sum up, increasing the expression level of circFOXO3 could inhibit the proliferation and migration of HCC cells, which may be achieved by inhibiting the PI3K/Akt pathway. However, the mechanism of proliferation and migration of HCC cells is complex, and circFOXO3 may inhibit the proliferation and migration of HCC cells in other ways. Therefore, further research is needed.

## Figures and Tables

**Table 1:** Comparison of expression levels of circFOXO3 mRNA between human hepatoma cells and normal hepatocytes ($\bar{x}\pm s$, *n* = 3).

Cell line	circFOXO3/U6
HHL-5	1.05 ± 0.18^a^
BEL-7404	0.60 ± 0.09^a^
Hep G2	0.59 ± 0.07^a^
Hep 3B2.1–7	0.62 ± 0.10^a^
HuH-7	0.44 ± 0.08^a^
Li-7	0.51 ± 0.08^a^

Compared with HHL-5, ^*a*^*P* < 0.05.

**Table 2:** Comparison of the expression level of circFOXO3 in HuH-7 hepatoma cells of each group ($\bar{x}\pm s$, *n* = 3).

Group	circFOXO3/U6
Control group	0.95 ± 0.13
circFOXO3-NC group	0.96 ± 0.14
circFOXO3 mimic group	1.67 ± 0.20^ab^
PI3K activator group	0.94 ± 0.17^c^
circFOXO3 mimic + PI3K activator group	1.69 ± 0.24^abd^

Compared with the control group, ^*a*^*P* < 0.05; compared with the circFOXO3-NC group, ^*b*^*P* < 0.05; compared with the circFOXO3 mimic group, ^*c*^*P* < 0.05; compared with the PI3K activator group, ^*d*^*P* < 0.05.

**Table 3:** Comparison of viability and apoptotic ability of HuH-7 hepatoma cells in each group ($\bar{x}\pm s$, *n* = 3).

Group	Cell viability (%)	Apoptosis rate (%)	Expression level of apoptosis-related proteins		
			Caspase-3/GAPDH	Bax/GAPDH	Bcl-2/GAPDH
Control group	100.00 ± 0.00	19.27 ± 2.32	0.97 ± 0.08	1.02 ± 0.15	0.97 ± 0.14
circFOXO3-NC group	72.39 ± 4.47	19.08 ± 2.29	0.95 ± 0.09	1.01 ± 0.12	1.01 ± 0.15
circFOXO3 mimic group	31.20 ± 2.37^ab^	48.37 ± 4.95^ab^	1.71 ± 0.19^ab^	1.72 ± 0.21^ab^	0.23 ± 0.01^ab^
PI3K activator group	125.27 ± 4.95^abc^	13.98 ± 1.33^abc^	0.63 ± 0.07^abc^	0.60 ± 0.06^abc^	1.46 ± 0.23^abc^
circFOXO3 mimic + PI3K activator group	54.82 ± 3.11^abcd^	34.40 ± 3.88^abcd^	1.32 ± 0.15^abcd^	1.31 ± 0.14^abcd^	0.57 ± 0.05^abcd^

Compared with the control group, ^*a*^*P* < 0.05; compared with the circFOXO3-NC group, ^*b*^*P* < 0.05; compared with the circFOXO3 mimic group, ^*c*^*P* < 0.05; compared with the PI3K activator group, ^*d*^*P* < 0.05.

**Table 4:** Expression level of PI3K/Akt pathway-related proteins in HuH-7 hepatoma cells in each group ($\bar{x}\pm s$, *n* = 3).

Group	p-PI3K/PI3K	p-Akt/Akt
Control group	1.02 ± 0.15	1.01 ± 0.14
circFOXO3-NC group	0.97 ± 0.14	0.98 ± 0.15
circFOXO3 mimic group	0.24 ± 0.03^ab^	0.26 ± 0.03^ab^
PI3K activator group	1.54 ± 0.25^abc^	1.55 ± 0.24^abc^
circFOXO3 mimic + PI3K activator group	0.59 ± 0.08^abcd^	0.63 ± 0.06^abcd^

*Compared with the control group,* ^*a*^ *P* < 0.05*; compared with the circFOXO3-NC group,*^*b*^*P* < 0.05*; compared with the circFOXO3 mimic group,*^*c*^*P* < 0.05*; compared with the PI3K activator group,*^*d*^*P* < 0.05.

**Table 5:** Comparison of invasion and migration abilities of HuH-7 cells in different groups.

Group	Number of invasive cells (number)	Scratch healing rate (%)	Expression level of invasion-related proteins	
			MMP-2/GAPDH	MMP-9/GAPDH
Control group	115.48 ± 10.03	60.43 ± 7.69	0.98 ± 0.09	0.97 ± 0.14
circFOXO3-NC group	115.06 ± 10.01	61.06 ± 7.35	0.96 ± 0.07	1.02 ± 0.11
circFOXO3 mimic group	60.10 ± 5.50^ab^	34.67 ± 3.78^ab^	0.65 ± 0.08^ab^	0.62 ± 0.05^ab^
PI3K activator group	150.26 ± 15.27^abc^	85.44 ± 13.05^abc^	1.73 ± 0.16^abc^	1.75 ± 0.20^abc^
circFOXO3 mimic + PI3K activator group	84.85 ± 8.15^abcd^	47.48 ± 4.20^abcd^	1.31 ± 0.13^abcd^	1.33 ± 0.12^abcd^

Compared with the control group, ^*a*^*P* < 0.05; compared with the circFOXO3-NC group, ^*b*^*P* < 0.05; compared with the circFOXO3 mimic group, ^*c*^*P* < 0.05; compared with the PI3K activator group, ^*d*^*P* < 0.05.

## Data Availability

The data generated or analyzed during this study are included within the article.

## References

[1] Bray F., Ferlay J., Soerjomataram I., Siegel R. L., Torre L. A., Jemal A. (2018). Global cancer statistics 2018: globocan estimates of incidence and mortality worldwide for 36 cancers in 185 countries. *CA: A Cancer Journal for Clinicians*.

[2] Fu J., Wang H. (2018). Precision diagnosis and treatment of liver cancer in China. *Cancer Letters*.

[3] Xiong Q., Bai Y., Shi R. (2021). Preferentially released mir-122 from cyclodextrin-based star copolymer nanoparticle enhances hepatoma chemotherapy by apoptosis induction and cytotoxics efflux inhibition. *Bioactive Materials*.

[4] Fibbe W. E., Shi Y. (2019). Foxo3, a molecular search for the fountain of youth. *Cell Stem Cell*.

[5] Yang W., Du W. W., Li X., Yee A. J., Yang B. B. (2016). Foxo3 activity promoted by non-coding effects of circular rna and Foxo3 pseudogene in the inhibition of tumor growth and angiogenesis. *Oncogene*.

[6] Takahashi M., Fukuda K., Saikawa Y. (2020). Role of Foxo3a in trastuzumab combination chemotherapy in esophageal squamous cell carcinoma. *Anticancer Research*.

[7] Xu F., Zhu F., Wang W., Gao W., Chen X., Yu C. (2020). Down-regulation of mirna-196b expression inhibits the proliferation, migration and invasiveness of Hepg2 cells while promoting their apoptosis via the pi3k/akt signaling pathway. *Cellular and Molecular Biology*.

[8] Wang W., Dong X., Liu Y. (2020). Itraconazole exerts anti-liver cancer potential through the wnt, pi3k/akt/mtor, and ros pathways. *Biomedicine & Pharmacotherapy*.

[9] Zhou Y., Li Y., Xu S. (2020). Eukaryotic elongation factor 2 kinase promotes angiogenesis in hepatocellular carcinomaviaPI3K/Akt and STAT3. *International Journal of Cancer*.

[10] Nguyen R., Bae S. D. W., Qiao L., George J. (2021). Developing liver organoids from induced pluripotent stem cells (ipscs): an alternative source of organoid generation for liver cancer research. *Cancer Letters*.

[11] Liu C. Y., Chen K. F., Chen P. J. (2015). Treatment of liver cancer. *Cold Spring Harbor perspectives in medicine*.

[12] Lee Y. S., Jeong S., Kim K. Y. (2021). Honokiol inhibits hepatoma carcinoma cell migration through downregulated cyclophilin B expression. *Biochemical and Biophysical Research Communications*.

[13] Yuan C., Luo X., Zhan X., Zeng H., Duan S. (2020). Emt related circular rna expression profiles identify Circscyl2 as a novel molecule in breast tumor metastasis. *International Journal of Molecular Medicine*.

[14] Zhang Y., Zhao H., Zhang L. (2018). Identification of the tumor‑suppressive function of circular RNA FOXO3 in non‑small cell lung cancer through sponging miR‑155. *Molecular Medicine Reports*.

[15] Xing Y., Zha W. J., Li X. M. (2020). Circular RNA circ‐Foxo3 inhibits esophageal squamous cell cancer progression via the miR‐23a/PTEN axis. *Journal of Cellular Biochemistry*.

[16] Wang Y., Xie Z., Lu H. (2020). RETRACTED: significance of halofuginone in esophageal squamous carcinoma cell apoptosis through HIF-1*α*-FOXO3a pathway. *Life Sciences*.

[17] Huang Y. Y., Jiang H. X., Shi Q. Y. (2020). miR-145 inhibits Th9 cell differentiation by suppressing activation of the PI3K/Akt/mTOR/p70S6K/HIF-1*α* pathway in malignant ascites from liver cancer. *OncoTargets and Therapy*.

[18] Li J., Zeng T., Tang S. (2021). Medical ozone induces proliferation and migration inhibition through ROS accumulation and PI3K/AKT/NF-*κ*B suppression in human liver cancer cells in vitro. *Clinical and Translational Oncology*.

[19] Zhangyuan G., Wang F., Zhang H. (2020). Versicanv1 promotes proliferation and metastasis of hepatocellular carcinoma through the activation of egfr-pi3k-akt pathway. *Oncogene*.

[20] He J. Q., Zheng M. X., Ying H. Z. (2020). Prp1, a heteropolysaccharide from platycodonis radix, induced apoptosis of Hepg2 cells via regulating mir-21-mediated pi3k/akt pathway. *International Journal of Biological Macromolecules*.

